# A case study of pulmonary embolism from the right atrial shunt after acute type a aortic dissection surgery

**DOI:** 10.1186/s13019-014-0180-y

**Published:** 2014-11-18

**Authors:** Wei J Yang, Qun J Duan, Hai F Cheng, Ai Q Dong

**Affiliations:** Department of cardiovascular surgery, The Second Affiliated Hospital of Zhejiang University School of Medicine, No. 88 Jiefang road, Hangzhou, China

**Keywords:** Aortic dissection, Postoperative complications, Pulmonary embolism

## Abstract

This is one case report of a 46-year-old Chinese male with type A acute aortic dissection. It is an emergent surgery including Bentall procedure, interposition graft replacement of aortic arch, stented descending aorta with the modified right atrial shunt technique. In the early post operation period, the patient was complicated with pulmonary embolism. Pulmanary arteriography showed that the right main pulmonary embolism without an extrinsic compression. After the anticoagulant therapy, the patient was well recovered and discharged from hospital 1 month later. The origin of pulmonary embolism in the patient was believed to be the thrombosis in the shunt fistula. The right atrial shunt-related complications haven't been reported for more than thirty years. Pulmonary embolism could be a severe complication after the right atrial shunt in acute type A aortic dissection.

**Electronic supplementary material:**

The online version of this article (doi:10.1186/s13019-014-0180-y) contains supplementary material, which is available to authorized users.

## Background

In the aortic root replacement procedure, bleeding from the proximal anastomosis is one of the gravest complications. A study showed that 5%-10% patients died of bleeding [1]. Cabrol first applied right atrial shunt to reduce bleeding in 1978 [2], in which a fistula between the aneurysmal sac and the right atrial appendage was created to drain oozing from the prosthesis. When bleeding from the proximal anastomosis was reduced, the thrombosis was formed, and then the shunt passage was closed up. Generally, the forming of thrombosis in the fistula is satisfied [3]-[5]. The shunt settled the problem of bleeding in the aortic root replacement procedure, and shortened the operating time significantly. The right atrial shunt-related complications haven’t been reported in more than thirty years. However, the patient in our case study was complicated with pulmonary embolism post operation, and there was very likelihood that the embolism was from this fistula.

## Case presentation

**Figure 1:**
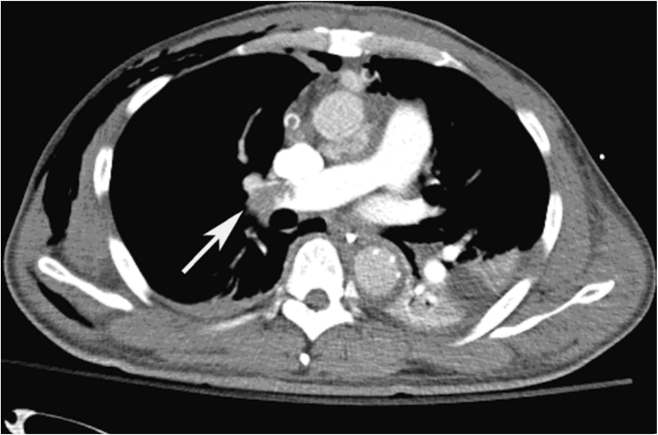
**The Pulmonary Arteriography on the fourth day post operation.** It showed that an entire right lung field defected near the crotch (arrow indicates pulmonary embolism).

**Figure 2:**
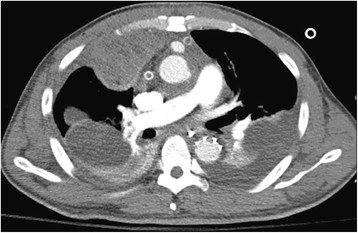
**The Pulmonary Arteriography on the twelfth day post operation.** It showed that the right lung field open again, and pleural effusion happened.

A 46-year-old Chinese male with a history of hypertension presented with sudden syncope and came to his senses after ten minutes without headache. His respiratory rate was 21 breaths per min and the lungs were clear on auscultation. Cardiac examination revealed a grade II/6 diastolic murmur at the parasternal border. The pulse rate was 75 beats per min with normal sinus rhythm. The blood pressure was 109/76 mmHg in the left arm and 112/62 mmHg in the right arm. No remarkable abnormal was found in abdominal examination. Coagulation tests were normal. Arterial gas analysis on 3 L/min oxygen inspiration was pH 7.39, PaCO_2_ 39.8 mmHg, PaO_2_ 62.5 mmHg, SaO_2_ 91.1%. A chest x-ray revealed widening of the mediastinum, a cardiothoracic ratio of 60%, and normal lung fields. Echocardiography revealed ascending aortic dissection and moderate aortic regurgitation with small to moderate pericardial effusion. Computed tomography scanning revealed ascending aortic to aortic arch dissecting aneurysm, in which the proximal part of right brachiocephalic trunk was also influenced. The left and right pulmonary arteries were normally enhanced with contrast medium. The patient underwent emergency operation for the thoracic aortic dissection, including Bentall procedure, interposition graft replacement of aortic arch, stented descending aorta with a shunt from a chamber around the aortic root to the right atrium. The patient was successfully weaned off bypass and transferred to the intensive care unit. The ratio of PaO_2_ to FiO_2_ after operation was 300. The haemodynamics was stable. However, the ratio of PaO_2_ to FiO_2_ fell to 150 gradually after operation. Two days after operation, the patient was conscious and cooperative, and his lung compliance and airway resistance were normal. But severe hypoxaemia ensued. In order to find out the cause of hypoxaemia, a chest computer temography was carried out but no evidence of atelectasis or pneumonia was shown. Pulmanary arteriography showed that an entire right lung field was defected without an extrinsic compression, which indicated embolism of the right pulmonary artery (Figure 1). Considering the stable haemodynamics and the danger of haemorrhage, embolectomy and thrombolytic therapy were not applied in the patient. We took the anticoagulant therapy with low molecular weight heparin and warfarin**,** which sustained INR at 2-3. Pulmanary arteriography was reexamined every 3-4 day. The images indicated that thrombus gradually dissolved (Figure 2). And the patient’s hypoxemia was improved. According to the effectiveness of treatment, we made sure that the entire right lung field defect in pulmanary arteriography was due to thromboembolism. Unfortunately, ventilator-associated pneumonia (VAP) occurred. Therefore, the tracheotomy tube was extubated until one month after operation. He survived for one year after discharging from the hospital without complications related to lung and heart.

## Conclusions

Acute aortic dissection (AAD) is the most common aortic catastrophe and is associated with extremely high morbidity and mortality. The mortality rate of type A dissection approaches 1% per hour and 40% to 50% in 48 hours without surgical therapy [6],[7]. However, in-hospital surgical mortality for acute type A aortic dissection was about 25% [8],[9]. Pulmonary embolism is a relatively common cardiovascular emergency, which may lead to acute life-threatening but potentially reversible right ventricular failure by occluding the pulmonary arterial bed [10]. When the patient of type A acute aortic dissection is complicated with pulmonary embolism, the prognosis may be even worse.

In most cases pulmonary embolism is a consequence of deep vein thrombosis. The patient in our case report was a middle-aged man without history of bone fraction, deep vein thrombosis or long-term bed pre-operation. Both lower extremities of the patient weren’t edema post operation, and dopplers ultrasonic examination was negative. Therefore, all these clinical features didn’t show deep vein thrombosis.

**Figure 3:**
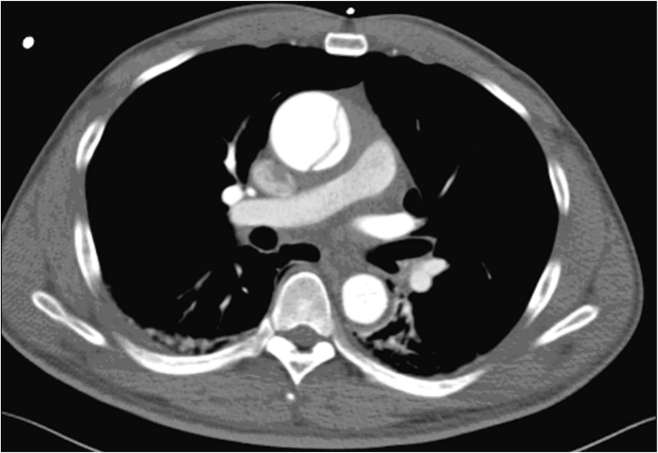
**The Aortic Arteriography before operation.** It showed that pulmonary artery was free without extrinsic compression or pulmonary embolism.

In 1972 L. Buja described that severe obstruction of the right main pulmonary artery as a consequence of acute dissection of the ascending aorta, appearing to have resulted from compression of this artery [11]. Case reports were reported with imaging data or autopsy results later [12]-[15]. These reports confirmed that the dilated ascending aortic or the periaortic haematoma compressed the right pulmonary artery. So the blood flow in pulmonary artery was slow and it was susceptible to thrombosis. However, in this case, CT images revealed that there was a long way off between the ascending aorta and the right main pulmonary artery pre or post-operation (Figures 1 and 3).

After exclusion of other possible factors, the origin of pulmonary embolism in the patient was believed to be the thrombosis in the shunt chamber. In order to reduce the bleeding post operation, a chamber around the aortic root was created by suturing residual aortic piece and patch of autologous pericardium, then the chamber was communicated with the right atrium to drain the blood from the proximal anastomosis. Dopplers ultrasonic examination showed no flow in the chamber four days post operation. A conclusion was drawn that pulmonary embolism from the thrombus of shunt chamber could be a severe complication after the right atrial shunt in acute type A aortic dissection.

## Consent

Written informed consent was obtained from the patient for publication of this case report and accompanying images. A copy of the written consent is available for review by the Editor-in-Chief of this journal.

## Authors’ contributions

WJY contributed in pre-operative and post-operative care. She also collected data and participated directly in the writing of the manuscript. QJD contributed in reoperative, intraoperative and post-operative care. He also revised the article thoroughly and critically. HFC contributed in preoperative, intraoperative and post-operative care. AQD contributed in preoperative, intraoperative and post-operative care. All authors approved the final version to be published.

## Authors’ original submitted files for images

Below are the links to the authors’ original submitted files for images. Authors’ original file for figure 1 Authors’ original file for figure 2 Authors’ original file for figure 3

## Abbreviations

AAD: Acute aortic dissection
CT: Computer tomography
PaCO_2_: Partial pressure of carbon dioxide in artery
PaO_2_: Arterial oxygen partial pressure
SaO_2_: Arterial oxygen saturation
FiO_2_: Fraction of inspiration oxygen
INR: International normalized ratio

## References

[1] Muehrcke DD, Szarnicki RJ (1989). Use of pericardium to control bleeding after ascending aortic graft replacement. Ann Thorac Surg.

[2] Cabrol C, Pavie A, Gandjbakhch I, Villemot JP, Guiraudon G, Laughlin L, Etievent P, Cham B (1981). Complete replacement of the ascending aorta with reimplantation of the coronary arteries: new surgical approach. J Thorac Cardiovasc Surg.

[3] Cabrol C, Pavie A, Mesnildrey P, Gandjbakhch I, Laughlin L, Bors V, Corcos T (1986). Long-term results with total replacement of the ascending aorta and reimplantation of the coronary arteries. J Thorac Cardiovasc Surg.

[4] Vogt PR, Akinturk H, Bettex DA, Schmidlin D, Lachat ML, Turina MI (2001). Modification of surgical aortoatrial shunts for inaccessible bleeding in aortic surgery – modification of the Cabrol-shunt technique. Thorac Cardiovasc Surg.

[5] Song MH, Tokuda Y, Nakayama T, Hattori K (2008). A simple method of inspection of proximal bleeding in Bentall procedure. Asian Cardiovasc Thorac Ann.

[6] Hines G, Dracea C, Katz DS (2011). Diagnosis and management of acute type A aortic dissection. Cardiol Rev.

[7] Feldman M, Shah M, Elefteriades JA (2009). Medical management of acute type A aortic dissection. Ann Thorac Cardiovasc Surg.

[8] Campbell-Lloyd AJ, Mundy J, Pinto N (2010). Contemporary results following surgical repair of acute type a aortic dissection (AAAD): a single centre experience. Heart Lung Circ.

[9] Trimarchi S, Nienaber CA, Rampoldi V (2005). Contemporary results of surgery in acute type A aortic dissection: The International Registry of Acute Aortic Dissection experience. J Thorac Cardiovasc Surg.

[10] Torbicki A, Perrier A, Konstantinides S (2008). Guidelines on the diagnosis and management of acute pulmonary embolism: the Task Force for the Diagnosis and Management of Acute Pulmonary Embolism of the European Society of Cardiology (ESC). Eur Heart J.

[11] Buja LM, Ali N, Fletcher RD (1972). Stenosis of the right pulmonary artery: a complication of acute dissecting aneurysm of the ascending aorta. Am Heart J.

[12] Nasrallah A, Goussous Y, El-Said G (1975). Pulmonary artery compression due to acute dissecting aortic aneurysm: clinical and angiographic diagnosis. Chest.

[13] Kutcher WL, Kaufman BS (1988). Occlusion of the right pulmonary artery by an acute dissecting aortic aneurysm. Crit Care Med.

[14] Masuo M, Takano H, Takamoto S, Tanaka S, Kitamura S, Saito T (2004). Pulmonary artery obstruction caused by thoracic aortic dissection: a case with unique pathological findings. Circ J.

[15] De Silva RJ, Hosseinpour R, Screaton N (2006). Right pulmonary artery occlusion by an acute dissecting aneurysm of the ascending aorta. J Cardiothorac Surg.

