# Supplementary figures and images for: A case study of pulmonary embolism from the right atrial shunt after acute type a aortic dissection surgery
Source: J Cardiothorac Surg. 2014 Nov 18;9:180. doi: 10.1186/s13019-014-0180-y (PMC4236451; doi:10.1186/s13019-014-0180-y)

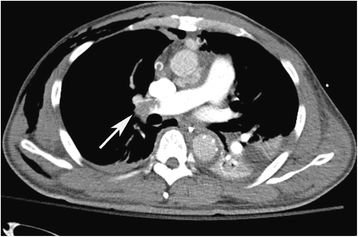

Supplement: Supplementary file 1 — Authors’ original file for figure 1 [file 13019_2014_180_MOESM1_ESM.gif]

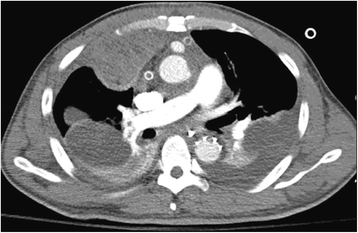

Supplement: Supplementary file 2 — Authors’ original file for figure 2 [file 13019_2014_180_MOESM2_ESM.gif]

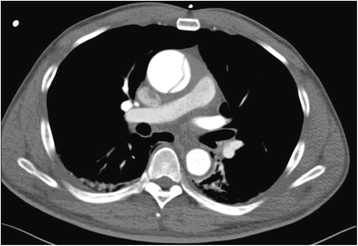

Supplement: Supplementary file 3 — Authors’ original file for figure 3 [file 13019_2014_180_MOESM3_ESM.gif]
